# Evaluation of a community dental clinic providing care to people experiencing homelessness: A mixed methods approach

**DOI:** 10.1111/hex.13111

**Published:** 2020-08-05

**Authors:** Martha Paisi, Rebecca Baines, Christina Worle, Lyndsey Withers, Robert Witton

**Affiliations:** ^1^ Peninsula Dental Social Enterprise Peninsula Dental School University of Plymouth Plymouth UK; ^2^ Well Connected (Charity) University of Plymouth Plymouth UK

**Keywords:** access, adults, dental care, homeless persons, oral health

## Abstract

**Background:**

People who experience homelessness have higher dental treatment needs compared to the general population. However, their utilization of dental services and levels of treatment completion are low. Peninsula Dental Social Enterprise, a not‐for‐profit organization in the United Kingdom, established a community dental clinic to improve access to dental care for this population.

**Objectives:**

To evaluate the impact and acceptability of the community dental service for patients and examine the barriers and enablers to using and providing the service.

**Methods:**

The evaluation included a retrospective assessment of anonymous patient data and thematic analysis of semi‐structured interviews with patients, support staff and service providers. The interviews were thematically analysed. A cost analysis of the dental service was also conducted.

**Results:**

By 18 February 2020, 89 patients had attended the clinic. These included 62 males (70%) and 27 females (30%), aged 38.43 years on average (SD ± 11.07). Of these, 42 (47%) patients have completed their treatment, 23 (26%) are in active treatment and 24 (27%) left treatment. In total, 684 appointments (541.5 hours clinical time) were given. Of these, 82% (562) of appointments were attended (452.5 hours clinical time). The 22 interviews that were conducted identified flexibility, close collaboration with support services and health‐care team attitudes as key factors influencing service utilization and continuity of care.

**Conclusions:**

This study provides details of a highly acceptable and accessible dental care model for people experiencing homelessness, with recommendations at research, practice and commissioning levels.

**Patient or Public Contribution**

- Potential patients, peer advocates with lived experience of homelessness and community care‐givers were involved in the design of the service evaluated in this paper.
- Patients and community care‐givers were interviewed as part of this study.
- A community care‐giver also contributed to the interpretation of data, as part of critically revising the manuscript.

## 1 INTRODUCTION

Homelessness is associated with increased morbidity and mortality.^1^ Dental problems are among the most common health concerns affecting people experiencing homelessness,^2^, ^3^ with higher levels of untreated dental disease and more missing teeth than the general population,^4^, ^5^ causing poorer oral health‐related quality of life.^6^ Severely limited access to dental care is compounded by high levels of non‐attendance and low levels of treatment completion.^7^, ^8^, ^9^ Both the lived experience of homelessness and characteristics of the health‐care system contribute to the low uptake of dental services.^10^

Disproportionate differences in oral health between population groups are due to an interaction of a number of factors (eg socioeconomic and political environment), many beyond an individual’s control.^11^, ^12^ Dental service utilization contributes to oral health inequalities.^13^ Watt and colleagues state that addressing this requires ‘coordinated strategic action at both clinical and population levels’.^12^ Freeman and colleagues^14^ have developed a theoretical framework for ‘inclusion oral health’ focusing on innovative solutions to tackle inequalities associated with poor oral health in individuals experiencing social exclusion. Their action plan addressing oral health services, research and dental education can make dentistry a powerful catalyst to reduce inequalities.^14^

Clearly, dental teams and services have a key role in improving ‘access and the quality of dental care for vulnerable groups’, acting as ‘advocates for policy change’ to reduce oral health inequalities.^12^ This is reflected in the UK National Health Service (NHS) long‐term plan, which prioritises the health care of those with additional needs.^15^ This plan also highlights the important role social enterprises play in addressing health‐care needs,^15^ since they can respond more flexibly to patients than other NHS bodies. The need for flexible service provision accommodating the complex needs of people experiencing homelessness is consistently highlighted in existing literature as strongly influencing utilization.^7^, ^9^, ^16^

Peninsula Dental Social Enterprise (PDSE) is a not‐for‐profit organization responsible for running the dental education clinics of the Peninsula Dental School, University of Plymouth. It is committed to improving oral health and reducing inequalities in the South West of the UK, through education, community engagement, training and treatment.^17^ One of its main aims is to ensure access to dental care for all, particularly those excluded from mainstream dentistry,^18^ including the homeless community. In response to the significant NHS dental waiting list in Plymouth city (over 14 000 people)^19^ and repeated calls for improved access,^20^ PDSE established a community dental clinic in January 2018 for those experiencing homelessness. PDSE’s approach lies within Freeman and colleagues’ inclusion oral health framework, which suggests that ‘*dentistry could act as an agent for social inclusion as a more responsive, all‐encompassing form of oral healthcare and delivery’*.^14^

Despite the acknowledged importance of such clinics, research exploring their impact and/or effectiveness has been limited, and mostly descriptive or quantitative. An important missing element is exploration of care models and processes that support or inhibit the delivery and use of dental clinics for individuals experiencing homelessness.^7^, ^9^, ^21^

### 1.1 Aim

This research aims to describe a care model developed for people who experience homelessness, evaluate its impact and acceptability from a patient perspective and examine the barriers and enablers to providing and using the service.

### 1.2 Description of model

The PDSE community dental clinic is located at the Dental Education Facility in Devonport, one of the most deprived areas in Plymouth.^22^ It is currently a pro bono contribution to the local community. Initially, the clinic treated people experiencing homelessness, expanding within the last year to include individuals using drug and alcohol services, as well as vulnerable women who risk of having multiple children removed from their care.

The first patients were triaged through a student project conducted in a residential homeless centre.^23^ Later, referrals were made through the ‘Teeth Matter’ oral health intervention project,^24^ the research findings of which have informed the development and running of the service.^24^, ^25^ Thereafter, referrals have been made primarily through the lead volunteer of the homeless centre and through support workers based in the other organizations that PDSE collaborates with. The lead volunteer, acting as a link worker, also facilitates referrals from a GP outreach service. She has 10 years of experience in the homeless sector, concentrating on health issues.

**Figure 1:**
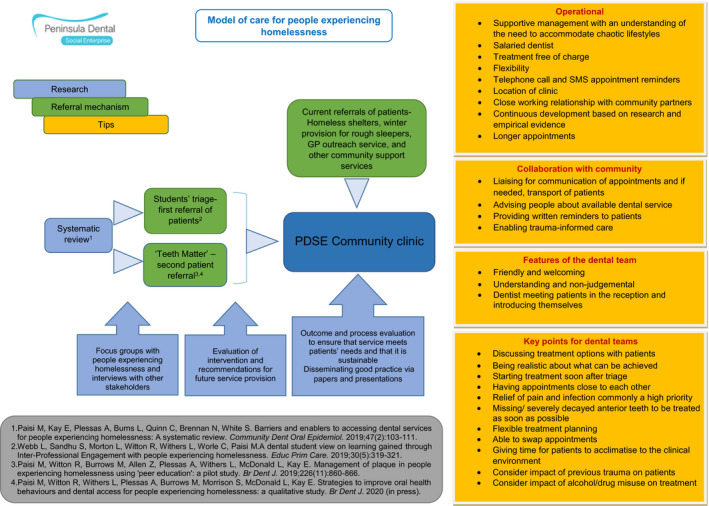
PDSE model of care for people experiencing homelessness

The community clinic began operating in January 2018, for half a day per week. This increased to a full day in August 2018 and then, due to high demand and success, two days per week in September 2019. A salaried dentist provides both routine and urgent treatment. Subject to patients’ consent, appointments are arranged in coordination with the lead volunteer or a support worker, who also provide appointment reminders, transport to the clinic and chaperoning during treatment, as needed. The model of care is presented in Figure 1.

## 2 METHODS

The evaluation included a retrospective assessment of anonymized patient data and semi‐structured interviews conducted with patients, support staff (a support worker and a volunteer), as well as service providers. A basic cost analysis of the service was also conducted.

### 2.1 Retrospective data analysis

The retrospective analysis focused on patient demographics, attendance figures, number and type of treatments provided and treatment status (complete/incomplete).

### 2.2 Qualitative research

#### 2.2.1 Theoretical approach

A phenomenological approach^26^ was adopted to study the lived experiences of patients, care providers and support staff in receiving, providing or facilitating care at the clinic.

#### 2.2.2 Design

This was a qualitative research study.

#### 2.2.3 Recruitment

Participants recruited using purposeful sampling were approached through a gatekeeper. Other stakeholders including support staff and service providers (managers, administrators and clinicians) were invited via email. Participants were given the opportunity to ask questions and signed consent forms prior to being interviewed.

#### 2.2.4 Setting

Patients and support staff were interviewed at the residential homeless centre. Service providers were interviewed at PDSE’s clinic premises.

#### 2.2.5 Data collection

Semi‐structured, face‐to‐face interviews were conducted from September 2018 to February 2020, until data saturation was achieved. Topic guides were used (Appendix S1) with the opportunity for participants to expand on issues important to them. The guides were informed by the findings of a systematic review and primary research conducted earlier by the research team.^10^, ^24^, ^25^ Most interviews (n = 19) were conducted by an academic researcher with extensive experience in patient and public involvement, including with people experiencing homelessness and mental illness [RB], but unknown to participants. Interviews lasted 20‐45 minutes were audio recorded and transcribed verbatim by another researcher.

### 2.3 Data analysis

A descriptive analysis of demographic and clinical data was conducted using IBM SPSS (version 24). Interview transcripts were uploaded onto NVivo 12 software (QSR International Pty Ltd. version 11, 2015), then thematically analysed [by RB]^27^ using an inductive approach. Reflective thematic analysis was chosen for its flexibility, and because it allows researchers to identify and interpret themes/patterns in a data set across different groups of people, leading to greater insight.^27^, ^28^ Following the six steps described by Braun and Clarke,^29^ a researcher [RB] immersed herself in the data, identified initial codes using a line‐by‐line approach, grouped the codes into themes and then reviewed the themes. The researcher then defined and named the themes and produced the report. To ensure rigour in the analysis rather than ‘correctness’ in the coding, a second experienced researcher [MP] reviewed the analysis and questioned how the data were coded, assumptions made, and the rationale for decisions.

### 2.4 Cost analysis

This is based on the NHS publicly funded model and includes the operating costs of the clinic to PDSE (based on pay and non‐pay costs) and the cost per case, with a comparison to NHS funding where the service to be formally commissioned. Community inputs, currently provided on a voluntary basis, were not costed.

### 2.5 Ethical approval

The study was reviewed and approved by the Faculty of Health Research Ethics and Integrity Committee, University of Plymouth (ref: 17/18‐854).

## 3 RESULTS

### 3.1 Participant demographics

At the time of writing (18 February 2020), 89 patients had attended the clinic. These included 62 males (70%) and 27 females (30%), aged on average 38.43 years (SD ± 11.07; range: 20‐65). The majority were British nationals (83, 93%). Patient characteristics are detailed in Table 1.

**Table 1:** Patient demographic characteristics

Demographic data	Number (%)
Gender	
Male	62 (70)
Female	27 (30)
Age	
Mean age	38.43
Range	20‐65
Ethnicity	
White British	82 (92)
White European	4 (5)
White Irish	1 (1)
Black and White Caribbean	1 (1)
Black African	1 (1)
Admission Route	
Residential homeless centre	56 (63)
Other homeless hostel	3 (3)
Drug and alcohol treatment service	5 (6)
Referral from student clinic	1 (1)
Homeless Outreach GP	4 (4)
Vulnerable women’s support centres	10 (11)
Homeless drop‐in day centre	5 (6)
Winter night shelter	5 (6)

Table 2 details the treatments provided. A total of 298 extractions took place, an average of three per patient.

**Table 2:** Type and number of treatments provided at PDSE clinic up to 18 February 2020

Treatment	Number of treatments completed (% by total treatments)	Number of Patients having this treatment type (% by total patients)
New patient examination	89 (9)	84 (94)
Oral hygiene instruction	105 (11)	74 (83)
Scale and polish	44 (5)	41 (46)
Periodontal treatment	29 (3)	29 (33)
Extractions	298 (32)	58 (65)
Fillings	224 (24)	47 (53)
Root canal treatment	3 (<1)	3 (<1)
Crowns	1 (<1)	1 (<1)
Partial dentures	38 (4)	25 (28)
Full dentures	28 (3)	17 (19)
Addition to, or relines of dentures	6 (1)	6 (7)
Recall examination	16 (2)	13 (15)

Table 3 provides information on treatment plan status, including reasons for discontinuing treatment. By 18 February 2020, 42 patients (47%) had completed a treatment plan, 23 (26%) were still in active treatment, while 24 patients (27%) had left treatment. There were no significant differences in gender (X^2^(1) = 0.77; *P* = .78), ethnicity (*P* = .78) or housing status (*P* = .190) between those who did or did not complete their treatment. However, those completing their treatment were significantly older (43.21, SD: 10.95 vs 34.13, SD: 9.28; *t* (64) = −3.42, *P* < .01).

**Table 3:** Status of treatment plans

Number of patients who have completed treatment plans	42 (47%)	
Number of patients with uncompleted treatment still in active treatment	23 (26%)	
Number of patients not in active treatment who have not completed treatment plans	24 (27%)	
Reason for not returning for treatment	Initially stopped attending but would like to return for more treatment	2 (2%)
	Referred for dental clearance under GA	1 (1%)
	Only wanted emergency treatment	1 (1%)
	Deceased	1 (1%)
	Imprisoned	2 (2%)
	Moved away for rehab	2 (2%)
	Left (city)	2 (2%)
	Contact Lost/Reason Unknown	13 (15%)

### 3.2 Cost analysis

The clinic offered 684 appointments (541.5 hours clinical time), of which 82% (562) were attended (452.5 hours clinical time). Fifty‐three of the 562 attended appointments (9.4%) included treatment for an urgent dental need. 17.8% (122) appointments were missed (excluding short notice cancellations), corresponding to 89 hours of clinical time. The average number of appointments attended per patient was 6 (4.7 hours clinical time), and an average of one appointment was missed per patient (0.9 hours clinical time).

The operating cost of the clinic to PDSE of £152.59 per hour includes pay costs (dentist and dental assistant) and non‐pay costs (clinic overheads, consumables, dental materials and laboratory costs). The average cost per course of treatment is £854.50. This compares unfavourably with the funding available (£300) from the NHS if the service were state funded only.

### 3.3 Qualitative research

From 22 interviews (nine PDSE staff members, 11 patients, one support worker, and one volunteer), key themes were identified and grouped within the following domains: barriers to accessing dental care in general; barriers to accessing and delivering the clinic; respective enablers; impacts of the clinic; and suggested improvements. The themes and sub‐themes are discussed below supported by verbatim interview extracts.

### 3.4 Barriers

#### 3.4.1 Barriers to accessing dental care in general

Patients and staff members identified a number of barriers including previous dental care experiences that ‘*create a fear based around attending and receiving treatment’* (support worker, participant 1), anxieties, a ‘*needle or dental phobia’* (staff, participant 2), and previous experiences of judgement or discrimination, for example ‘*I think they used to take one look at me and go ‘oh no chance’ and realised I needed so much work, shove me off down the road to the next one’*. (patient, participant 3) Many staff members attributed barriers to the ‘*way the NHS contract works, it disincentivises practices for taking on patients with high treatment needs and perhaps chaotic lives …’;* (staff, participant 4) ‘*unfortunately these patients are seen as undesirable for an NHS system and it’s not financially viable for dentists and practices to see them’*. (staff, participant 5) Other barriers included embarrassment and/or shame, issues of addiction, chaotic lifestyles and low literacy levels.

#### 3.4.2 Barriers to accessing the clinic

The main barrier described related to patient‐preparedness. One staff member identified the ‘*chaotic lifestyles’* (staff, participant 5) of patients. This often meant oral health was not ‘*at the top of their priority list’*. (staff, participant 6); ‘*we call these people chaotic and that’s a bit judgmental, they are actually setting priorities, they’ve got so much going on in their lives that it [oral health] just falls of their list of priorities, they’re saying ‘it’s my priority to find somewhere to sleep tonight’ … The time that you catch people’* was therefore identified as *‘really important’*. (volunteer, participant 7) No patients described any barriers to accessing the clinic.

#### 3.4.3 Barriers to delivering the clinic

Of the few barriers identified, most related to challenging behaviours attributed to ‘*severe mental illness’*, addiction and/or aggression. However, these were more things to consider than insurmountable barriers. For example, ‘*[the dentist] was initially a bit apprehensive about managing some of the patients … it can be intimidating sometimes when someone’s rocking around or shouting, or doesn’t seem to be listening but you have to see past that because that’s just the manifestation of underlying social, or medical issues’*. (staff, participant 4) Staff noted that ‘*Initially we were thinking ‘oh we need to make sure that we’re not alone in the surgery at any point’, and we had a panic alarm and things, we still have all that in place, but it’s actually been fine’*. (staff, participant 5).

Other barriers included the transient nature of patients, for example ‘*They may have gone back onto the streets, the hostel worker may have lost contact with the patient again’* and mobile phone numbers ‘*never stay the same for very long’*. (staff, participant 6) ‘*Some people have gone to prison halfway through treatment … sometimes they will be heading off to rehab’*. (staff, participant 5).

### 3.5 Enablers

#### 3.5.1 Enablers in accessing the service

##### Staff

The non‐judgemental, empathetic, ‘*friendly and helpful’* (patient, participant 8) approach of staff members was identified as integral in facilitating access: ‘*I didn’t feel judged at all’* (patient, participant 8); ‘*all the staff are really nice and helpful, I’ve had nothing but good experiences. … I felt treated like a normal person … it made me feel like I was worth something’* (patient, participant 9). ‘*Feeling cared for, and feeling that you deserve care is something that is often rare for these people’*. (volunteer, participant 7).

The dentist was frequently described as ‘*absolutely brilliant’* (patient, participant 3), due to her person‐centred approach, excellent ‘*interpersonal skills’* (volunteer, participant 7), ability to ‘*put you at ease’* (patient, participant 10), provision of ‘*positive feedback when people are brushing well’* (volunteer, participant 7), and clear, accessible explanations about treatment plans, options and progress.

##### Hostel (link) worker involvement

The involvement of a volunteer from the homeless hostel was also seen as ‘*essential’*. (staff, participant 11) The volunteer often helped broker introductions, provided encouraging support and, at times, transported patients to the clinic. Providers considered her integral in facilitating service delivery, reminding patients about appointments, maintaining patient contact, acting as a trusted source of information, providing background histories and chaperoning less confident patients. As favourably described by a number of participants: ‘*… it’s just the constant [volunteer] telling me ‘no, it’d be fine it’d be fine it’d be fine’ and just totally reassuring me and she took me down there on the first appointment’*. (patient, participant 12); ‘*attendance was an issue at first, but [volunteer] does try to attend appointments with the ones that don’t feel confident coming on their own; that’s made a difference’* (staff, participant 11); ‘*She’s got a relationship with each of them, she’s able to communicate with us and with them so it’s a three way thing, it works really well … I think the key thing is partnership and trust, … that community engagement element is really, really important’* (staff, participant 4) The importance of a ‘*community engagement’* model was repeatedly discussed by staff participants.

##### Chaperone

Patients also described friends who had chaperoned them to the clinic. While this was disruptive on a small number of occasions due to issues of intoxication and the number attending (which stopped after providing feedback), chaperones were largely identified as beneficial. For example, ‘*before I used to have to be sedated to actually go to the dentist, but my friend was with me all the time. Now I’ve got used to it, I go on my own’* (patient, participant 8) As a result, PDSE staff members have often made patients aware of the option to bring a chaperone, provided they are not disruptive to the clinic setting, highlighting the importance of communicating expectations as described below.

##### Patient readiness

Patient readiness or motivation was identified as key. For example, ‘*I know I had to get my teeth sorted out, I know I had to get it done’* (patient, participant 10); ‘*I know I needed it done and there’s no access to dental care anywhere else’* (patient, participant 9); ‘*you have to get someone at the right point of momentum’*. (staff, participant 5). Factors influencing readiness included pain, desperation, enhanced confidence following student/researcher contact, peer encouragement, a desire to get their smile back and aspirations of potential employment.

##### Reminders

Appointment stickers and text reminders were identified as beneficial in facilitating access and engagement: ‘*They gave me a couple of stickers with my dates on, but they would also text to remind me and that was brilliant because even though it’s stuck on my calendar right in front of me, having that text sort of kicked my head’* (patient, participant 8).

##### Longer appointment times and limited waiting times

Other factors found to encourage clinic attendance and engagement included longer appointment times to help make patients feel at ease, limited waiting times from registration to first appointment, brief waits in the reception area and brief intervals between appointments: ‘*I think that would be a killer if you had to wait, that would do me in because that gives you time to think, and if I’m thinking, I’m liable to do the off’* (patient, participant 3). Having appointments ‘*on the same day, at the same time’* (patient, participant 13) was also identified as beneficial as people were less likely to forget.

##### Student and research involvement, clinic location and environment

Many participants described the initial contact with students and research staff at the residential homeless centre, the clinic’s location (easy walking distance from the centre), its quiet, clean, non‐clinical smell and open environment as important in encouraging attendance and engagement: ‘*I think seeing people on home territory as a first encounter is great. We’ve got people into treatment who would never have walked into a dental surgery … it just broke down that barrier, a little quick check up that wasn’t too painful, a general quick triage assessment’*. (volunteer, participant 7). Patients agreed, appreciating the community engagement model, ‘*I felt more relaxed than going down there [to the clinic], if it [the triage] hadn’t been put in here, I wouldn’t have gone’*. (patient, participant 14) Holding the clinic on a day dedicated to patients experiencing homelessness was beneficial: ‘*It’s normally busy all the time and when a homeless person comes in there’s that stigma as soon as they walk [in], it’s full of people, so separating the clinics has helped them get through the door’* (staff, participant 15).

Continuity provided by ‘*appointments always being on a Monday’* (staff, participant 11) was seen as beneficial by both patients and staff. Use of a private surgery rather than bays in a larger multiple‐patient teaching surgery was appreciated by patients. It offered them the possibility of sharing their oral health history in private, recounting for example loss of teeth through a violent domestic abuse assault, or act of ‘self‐harm’ as disclosed by one participant.

#### 3.5.2 Enablers in delivering the service

##### Flexibility

Flexibility was identified as most influential enabler. This included allowing another patient to step in if the original patient could no longer attend, responding to patient circumstances that affected attendance in an understanding and supportive way, providing greater allowance for missed appointments than usually permitted and allowing patients to acclimatize to the clinic before any dental work starts.

##### Funding

The clinic’s funding structure was identified as significant: ‘*I’m paid a salary, I don’t work under the NHS UDA [payment method] system … [so] I can give a lot of time to people, I can be more flexible with them’*. (staff, participant 5) As noted earlier, many staff members acknowledged that ‘*the way the NHS contract works, disincentivises practices for taking on patients with high treatment needs and perhaps chaotic lives …’*; (staff, participant 4) ‘*… unfortunately these patients are seen as undesirable for an NHS system and it’s not financially viable for dentists and practices to see them’* (staff, participant 5).

##### Establishing clear boundaries

Managing patient and staff expectations regarding expected behaviour was considered key: ‘*Being very clear and upfront about what we expect from them [patients] is really important’* (staff, participant 4).

### 3.6 Impact of the clinic

Participants identified a number of patient benefits (Table 4). Outcomes were often described as a catalyst for change in multiple areas of a patient’s life: ‘*Emotionally he’s transformed, nutritionally he’s put on weight because he’s able to eat, his self‐esteem, his confidence and employment opportunities, his sense of worth is now fully established … his decision was ‘I either continue on this path of destruction’, which was very much influenced by his childhood experiences, or ‘I survive and thrive and I move forward’. And he chose the latter, and part of that was because he was linked to the Dental School’* (support worker, participant 1).

**Table 4:** Impact of dental treatment on patients

**Oral health impacts**	Improved oral hygiene	*‘Something happened to me, I stopped brushing – it was like a form of self‐harm, I just stopped brushing my teeth for a whole year, but now she’s [dentist] got me brushing twice, three times a day’* (patient, participant 17*); ‘Dental care is on the agenda here [homeless hostel] now, which is great’* (volunteer, participant 7)
**Physical health impacts**	Enhanced nutrition	*‘He was very drawn, very thin, it aged him, he would eat separately because of his inability to chew …’* (support worker, participant 1)
**Psychosocial impacts**	Improved confidence and self‐esteem	*‘It helps you get your confidence back’* (patient, participant 10); ‘*It’s given them the confidence that perhaps they didn’t have before to enable them to go on and try and better themselves’* (staff, participant 19).
	Getting a smile back	*‘I feel 100% better, I can smile again’* (patient, participant 9) ‘*when we fitted them [dentures] it changed her life completely, she couldn’t stop smiling’* (staff, participant 6)
	Happiness	*‘I haven’t got words to say how happy I am, it’s that last thing for me to get sorted out, to start my new life because I didn’t half get judged for bad teeth, people out in the shops notice and that, they’re all so happy for me because they saw how down and depressed I was all the time, I never smiled … it makes a lot of difference’* (patient, participant 8)
	Improved body image	*‘I’ve only just started to look in the mirror, I haven’t looked at myself in the mirror for fifteen years’* (patient, participant 8)
	Learning to trust health‐care professionals	*‘I’ve gone from someone that will actually physically harm a dentist* [through fear]*, to actually going because I started to trust, I learned how to trust’* (patient, participant 8)
**Economic impacts**	Employment	*‘It gave me so much more confidence, a lot’s come out of that, I mean the day I actually got my dentures I had a job interview and I just felt so much more comfortable and I actually got the job’* (patient, participant 9)
	Wider service engagement beyond the dental clinic	*‘it’s helped me get a job it’s helped me like move forward in society like training and everything so it’s meant a great deal towards my future’* (patient, participant 8)
	Career aspirations	*‘It’s made me think a bit broader on what I can do’* (patient participant 3)

Impacts were also identified at the staff level. PDSE staff members found the clinic ‘*very rewarding’, ‘humbling’* and ‘*worthwhile’* with some becoming emotional when discussing the impacts of the service. Several reported a change in their own attitudes, for example ‘*I think you’ve got a perception of what a homeless person may be like, they’re taking drugs etc. but actually you’re stereotyping, they’re just like me and you, they’ve just gone through a hard time, it’s been a real eye opener for all of us, it’s changed my perception really because I wouldn’t [now] be so judgmental’* (staff, participant 16).

### 3.7 Suggested improvements

Most patients identified no areas for improvement: ‘*I wouldn’t change a thing, I don’t think there’s any way you can improve it, I really sincerely mean that … it’s life changing’* (patient, participant 17).

Staff suggested the provision of aftercare, delivering ‘*more work in the hostel itself’* (staff, participant 4), providing a drop‐in clinic or mobile unit to facilitate access, involving GPs, providing other health‐care professionals with more information about the clinic and having the opportunity to provide positive patient feedback back to staff directly involved. However, implementing many of these suggestions would depend on securing sufficient funding: ‘*we need to attract funding* … *it's very difficult to encourage NHS England to commission outside of their routine, the existing contract doesn't favour patients with high treatment needs so we would need them to step outside of their comfort zone and commission something slightly different to what they're used to’* (staff, participant 4).

## 4 DISCUSSION

Our study has shown that the community dental clinic is highly successful in terms of uptake of care and subsequent attendance. It is positively perceived by patients, support staff and health‐care providers alike and has a significant positive impact on patients who demonstrate willingness to engage in treatment. Flexibility, close collaboration with support services and attitudes of the health‐care team strongly influence the utilization of the service, continuity of care and attendance rates.

We found that 42 out of 89 (47%) of patients had completed a treatment plan, while 27% failed to return for treatment completion. In contrast, previous research evaluating services for people experiencing homelessness^7^, ^9^, ^16^, ^21^ demonstrated lower levels of attendance and completion. A review of 204 patients attending a targeted dental service in London from 1992 to 2001, indicated that only 18% completed their treatment.^7^ A community clinic in Australia providing dental care to young people experiencing homelessness had a high percentage of failure‐to‐attend (57%) pre‐booked appointments.^21^ A unique feature of the care pathway developed by PDSE, and found to be crucial for the success of the clinic, is the role of a link worker. The benefit of having a bridge between patients and the service provides a sense of peer‐type to help vulnerable patients feel that treatment is within their reach, and it can also improve clinical time efficiency.

Link workers can make people aware of the availability of dental care, and their ‘insider knowledge’ of individual patients can enable the dental team to take patients’ circumstances into account and provide a truly patient‐centred service. Link workers can also assess whether a patient needs additional support to attend or to, for example, complete a medical history questionnaire. Moreover, they can notify the clinic of last minute cancellations due to a medical concern or other unforeseen issue and identify other patients who can make use of the appointment, avoiding lost clinical time. Thus, link workers can support service sustainability and patient satisfaction. Ideally, they should have experience in working with patients with complex needs and understand the importance of oral health to be able to motivate patients to seek treatment and help them keep their appointments.

For the approach to be fully effective, flexibility in service provision is essential, reflecting findings of previous studies.^9^, ^16^ High failure‐to‐attend among this population may be due to the inability of services to accommodate chaotic lifestyles.^16^ Thus, adapting to patients’ diverse needs is paramount in promoting uptake.

People with experience of homelessness commonly have a history of marginalization, at societal and health‐care service levels,^8^ compounded by a perceived stigma from health‐care teams^10^ which may exacerbate anxiety.^6^ Our findings demonstrate the importance of the dental team’s approach to patients’ dental journeys, with the attitudes and professionalism of both reception clinical staff being highly valued by patients. With regard to the dentist, the patients acknowledged how important it was for them to be able to discuss treatment plans and options. This approach helps patients feel empowered and actively involved in their treatment.

### 4.1 Implications

For patients with multiple and complex care needs, a reductionist approach is unlikely to work well, suggesting that expanding the professional network to facilitate integrated care with other health and social services would be advantageous. For example, many patients could benefit from wider support, for example nutrition, smoking cessation, blood‐borne virus issues, addiction management, mental health resilience. A more joined‐up approach and better communication among professionals could facilitate broader conversations about health care and help improve patients’ overall wellness.

The fact that providers’ attitudes changed positively over time highlights the significance of raising awareness among staff of the complexities of homelessness, breaking down barriers and challenging pre‐conceptions.

It is important for dental providers to recognize that people with complex needs most likely have experienced serious trauma and respond appropriately. This highlights the need for dental teams to receive training in managing patients with adverse childhood experiences and mental health and alcohol/substance misuse issues, to feel confident in providing trauma‐informed care (ie understanding the impact of trauma on an individual’s life and providing an environment where patients feel safe and can develop trust).^30^

Outreach programmes (either by students, health‐care professionals or researchers) at a location where people experiencing homelessness feel comfortable (eg residential homeless centre) can be used effectively to introduce people to oral health care and signpost them to available services. This can also inform the development of needs‐based services and help build relationships between patients and the dental teams. This could apply similarly to reducing inequalities in oral health care for other disadvantaged groups such as care leavers, asylum seekers and refugees, and victims of sexual abuse.

The PDSE service is provided free of charge to patients within a salaried service model. This provides the necessary flexibility for the complexities of the patient group and relies on a community‐supported care pathway for success. The significant cost discrepancy between state funding and the actual costs of providing care to this patient group means that the service would not be financially viable under a contract based on the current NHS dentistry payment method in England. New flexible models of care need to be developed that reward health‐care professionals appropriately to provide routine and continuing care for socially vulnerable adults with high treatment needs.

### 4.2 Strengths and weaknesses of the study

The study explored the views of patients, support staff and providers, offering insight into the views and experiences of all those involved. The use of thematic analysis enabled systematic analysis of the data. In order to ensure its trustworthiness,^28^ it is important to ensure the credibility, confirmability, dependability and transferability of the findings. Involvement of a second experienced researcher in the analysis of data ensured credibility. To attain confirmability, the narrative descriptions were supported by the relevant context and quotes so that findings could be trusted. To ensure dependability, the research and thematic analysis process was clearly documented.

The principles of success identified (flexibility, community‐supported pathway, relationships, trust, patient‐centred care and funding) would likely apply in any context and setting. However, given that operation and funding streams for dental health services differ between countries (eg public‐funded vs private dental care), the transferability of our findings to other homeless populations and dental systems may not always be feasible. By providing details about our context, others can judge the transferability of our findings to other contexts and populations.

The reasons for many dropouts, where known, have been provided. However, the transient nature of homelessness would have made tracking down other patients particularly challenging, although this could have given an insight into their attitudes and barriers to attendance, helping tailor services for this subgroup.

Lastly, when conducting thematic analysis, interpretation of findings is prone to the researcher’s subjectivity. For this reason, we have provided details on the researcher’s background.

### 4.3 Unanswered questions and future research

Identifying elements of a successful pathway that allows an increased integration of dentistry with other services is important and can lead to improved patient outcomes. For example, considering the high prevalence of tobacco use and alcohol consumption among the homeless community,^6^, ^8^ investigating the acceptability, feasibility and effectiveness of providing smoking and alcohol advice at a dental setting, is warranted.

Studies exploring the impact of ‘peer support’ by those who completed their dental treatment on encouraging uptake and maintenance of dental service use among other people experiencing homelessness are needed. This could help identify attributes sought in a ‘peer’ or ‘link worker’ to help promote uptake of care.

Conducting interviews with commissioners, to explore their views and attitudes, as well as challenges, towards flexible commissioning services for vulnerable groups, is recommended.

## 5 CONCLUSIONS

This study provides details of a highly successful, acceptable and accessible dental care model for people experiencing homelessness that could be implemented in other locations. It highlights the paramount importance of delivering a flexible service that accommodates the complex needs of this patient group, working closely with community services, treating patients with compassion and providing trauma‐informed care. Although successful in terms of patient and provider acceptability, it would be preferable in the interests of sustainability that future services be funded through flexible commissioning by the NHS.

## CONFLICT OF INTEREST

Robert Witton is the Chief Executive Officer of Peninsula Dental Social Enterprise. He did not participate in data analysis and interpretation. The other authors declare no conflict of interest.

## AUTHOR CONTRIBUTIONS

MP: Made substantial contributions to study conception and design, and acquisition, analysis and interpretation of data. Drafted the manuscript and revised it critically for important intellectual content. Gave final approval of the version to be published. Agreed to be accountable for all aspects of the work in ensuring that questions related to the accuracy or integrity of any part of the work are appropriately investigated and resolved. RB: Made substantial contributions to acquisition of data, analysis and interpretation of data. Involved in drafting the manuscript and revising it critically for important intellectual content. Gave final approval of the version to be published. Agreed to be accountable for all aspects of the work in ensuring that questions related to the accuracy or integrity of any part of the work are appropriately investigated and resolved. CW: Made substantial contributions to acquisition and analysis of quantitative data. Involved in revising the manuscript critically for important intellectual content. Gave final approval of the version to be published. Agreed to be accountable for all aspects of the work in ensuring that questions related to the accuracy or integrity of any part of the work are appropriately investigated and resolved. LW: Made substantial contributions to study design. Involved in revising the manuscript critically for important intellectual content. Gave final approval of the version to be published. Agreed to be accountable for all aspects of the work in ensuring that questions related to the accuracy or integrity of any part of the work are appropriately investigated and resolved. RW: Made substantial contributions to study conception and design. Involved in drafting the manuscript and revising it critically for important intellectual content. Gave final approval of the version to be published. Agreed to be accountable for all aspects of the work in ensuring that questions related to the accuracy or integrity of any part of the work are appropriately investigated and resolved.

## Supporting information

Appendix S1

## Data Availability

The data that support the findings of this study are available from the corresponding author upon reasonable request.
