# Supplementary material for: Evaluation of a community dental clinic providing care to people experiencing homelessness: A mixed methods approach
Source: Health Expect. 2020 Aug 5;23(5):1289–99. doi: 10.1111/hex.13111 (PMC7696139; doi:10.1111/hex.13111)
Supplement: Supplementary file 1 — Appendix S1 [file HEX-23-1289-s001.docx]

**Interview guide for support staff**

Gender:

Location:

Current position:

Years in this role:

Years working with people experiencing homelessness:

- Please can you tell me about your current responsibilities at […]?
- Has anything worked well with the […] service providing dental care to people experiencing homelessness? If yes, to specify.
- Are there any aspects of the service that you think have not worked as well/ could be improved?
- Were there any challenges/difficulties you encountered as a care provider of the patients during their dental journey? If yes, to specify
- Do you believe the visit by the school at [ ….] has had any impact on people’s attitude/feelings towards seeking dental treatment?
- Do you feel there were any differences between the first/second (Student project-March 2018 onwards) and third cohort of patients (research project-September 2018 onwards) that received treatment at […] clinic? (in terms of anxiety, punctuality, attitude towards dental treatment etc).
- Have you seen any changes in people’s attitudes towards dental care and/or feelings over the course of treatment?
- Overall, do you think the service is worthwhile? If yes, in what aspect? What were the key points of success?
- Do you have any recommendations for future dental service provision for people experiencing homelessness?
- Anything else to add?

**Interview guide for service providers**

Gender:

Current position:

Years in this role:

Years working with people experiencing homelessness

- Please can you tell me about your current responsibilities at […] and in what aspect you work with people experiencing homelessness?
- Has anything worked well with the new service providing dental care to people experiencing homelessness that made it easier for you to administer the service/deliver care? What were the key points of success?
- Were there any challenges/difficulties you encountered during the development and delivery of the service?
- Do you feel there were any differences between the first/second (Student project-March 2018 onwards) and third cohort of patients (research project-September 2018 onwards) that received treatment at […] clinic? (in terms of anxiety, punctuality, attitude towards dental treatment etc).
- Have you seen any changes in people’s attitudes towards dental care and/or feelings over the course of treatment?
- Have you experienced any changes in your own attitude understanding about homeless people?
- Overall, do you think the service is worthwhile? If yes, in what aspect?
- Is there anything you think could be improved?
- What insights have you gained from working on this dental service that would be useful to consider when developing a good practice model for people experiencing homelessness? Do you have any recommendations for future service provision? If you had to help design the service again, what would you keep and what would you change?
- Anything else to add?

**Interview guide for people experiencing homelessness**

Date of birth:

Nationality:

Gender:

Location:

How did you find out about the clinic?

- Please can you tell me what made you seek dental care?
- How did you initially feel about going to the dental clinic? (Explore reasons). How do you feel about going there now? (Explore reasons).
- Did the visit by the dental school to your place have any impact on your attitude towards seeking dental treatment?
- Have your attitudes towards dental care or the way you feel in general changed over the course of treatment? If yes, in what aspect?
- Have you been able to keep up your appointments? If not, why?
- Is there anything that you liked about the service overall? that helped you attend/use the service (e.g. dentist, receptionist, setting, location etc)?
- Is there anything that you did not like about the service or that has not worked well for you - that made it difficult for you to attend/use the service?
- Is there anything that helped you with attending the service and completing your treatment?
- Was the treatment you received important for you? Has the treatment you received impacted on your life or the way you feel in any way?
- Overall, do you think the service is worthwhile. If yes, in what aspect?
- If you had to design the service again, what would you keep and what would you change? Is there any way that the service could be improved?
- Is there anything you would like to add/recommend?
